# Dynamic Properties of the Solow Model with Bounded Technological Progress and Time-to-Build Technology

**DOI:** 10.1155/2014/908629

**Published:** 2014-03-19

**Authors:** Luca Guerrini, Mauro Sodini

**Affiliations:** ^1^Department of Management, Polytechnic University of Marche, 60121 Ancona, Italy; ^2^Department of Economics and Management, University of Pisa, 56124 Pisa, Italy

## Abstract

We introduce a time-to-build technology in a Solow model with bounded technological progress. Our analysis shows that the system may be asymptotically stable, or it can produce stability switches and Hopf bifurcations when time delay varies. The direction and the stability criteria of the bifurcating periodic solutions are obtained by the normal form theory and the center manifold theorem. Numerical simulations confirms the theoretical results.

## 1. Introduction

In one of the most influential papers on economic growth, Jones [1] argued that R&D-based models of growth à la Romer [2] are characterized by the counterfactual property that an increase of the level of resources invested on R&D sector implies an increase in the growth rate of economy. However, from empirical studies it appears clear that this result is inconsistent with time series evidence (see [3]) that suggests decreasing returns in the production of new technology, probably due to negative externalities (see [4]) or difficulties in creating new knowledge. Thus, in order to have a more realistic description of technological progress, Jones proposes an equation of accumulation with decreasing returns. The main finding of this approach is that the evolution of long-run economic growth is not endogenous but depends on classical factors usually taken as exogenous such as the rate of labor force. Given these styled facts, we introduce an exogenous growth model à la Solow [5] (thus, we do not model the allocative problem between research and manufacturing) in which technological progress is bounded from above and the rate of growth of the economy (i.e., capital accumulation) on the balanced growth path is equal to the rate of population growth. On the other hand, we introduce a time-to-build technology in which we assume the existence of a production lag that corresponds to the time for new capital to be produced and installed (see [6, 7]). The main objectives of the present paper are (i) to improve the understanding of the dynamic interaction between technological findings and the economic system; (ii) to characterize the possibility of self-sustained oscillations (business cycles phenomena) which are not possible in formulations without time delays (see [8, 9]).

From a mathematical point of view, it is worth mentioning that the characteristic equation associated to the dynamical system involves delay-dependent coefficients. Then, the corresponding dynamics are dramatically different with respect to models with delay independent parameters (e.g., [6, 10–15]) and stability switches as well as Hopf bifurcations may arise when time delay varies. In order to perform such analysis, we will use the procedure described in Beretta and Kuang [16] based on the existence of real zeros of particular functions *S*_*j*_(*τ*).

The paper is organized as follows. In Section 2, the model is described. In Section 3, by choosing the delay parameter as a bifurcation parameter and using the procedure introduced by Beretta and Kuang [16], we determine the condition for Hopf bifurcation occurrence for the model. In Section 4, an analysis on Hopf bifurcation including the direction and stability of bifurcation periodic solutions is made. In order to support the theoretical results, simulations are presented in Section 5. Some conclusions are drawn in Section 6.

## 2. The Mathematical Model

As explained in the introduction we are concerned with the modelling of à Solow model with bounded technological progress, where the Cobb-Douglas technology displays a delay of *τ* periods before capital can be used for production. Specifically, technological progress *A* evolves according to the *S*-shaped power law logistic technology [9]

(1) $$\begin{array}{c} \dot{A}=aA\left[1-{\left(\frac{A}{A_{*}}\right)}^{b}\right],\;0<A_{0}<A_{*}, \end{array}$$

where *A*_∗_ is the maximum level of technology and *a*, *b* are positive constants. The rate of change of the capital stock *K* at moment *t* is a function of the productive capital stock at *t* − *τ*; namely, we have $\dot{K}=sK_{d}^{\alpha }{\left(AL\right)}^{1-\alpha }-\delta K_{d}$, where *K* denotes physical capital, *K*_*d*_ = *K*(*t* − *τ*), *α* ∈ (0,1) is capital's share, *s* is the constant saving rate, and *L* represents labor force. As usual, population size and labor force are assumed to be interchangeable. Setting *k* = *K* / *AL*, the evolution of per capita physical capital over time is given by

(2) k˙=s[(AdA−1)(LdL−1)]αkdα−δ[(AdA−1)(LdL−1)]kd −(A˙A+L˙L)k.

Population grows at a constant rate *n* > 0. Hence, normalizing the number of people at time zero to one yields *L* = *e* ^*nt*^. Setting *h* = (*A* / *A*_∗_)^*b*^, so that $\dot{h}\;/\;h=b\dot{A}\;/\;A$, and by substituting the above equations, we can rearrange terms to have the model described by the following delay differential system with delay-dependent coefficients

(3) k˙=se−αnτ[(hdh−1)]α / bkdα−δe−nτ(hdh−1)1 / bkd −[n+a(1−h)]k,h˙=abh(1−h).

Letting $\dot{k}=0$, $\dot{h}=0$, *h*_*d*_ = *h*, and *k*_*d*_ = *k*, the equilibria of system (3) are determined. We derive that there exists a unique nontrivial equilibrium (*k*_∗_, *h*_∗_), where

(4) $$\begin{array}{c} se^{-\alpha n\tau }k_{*}^{\alpha -1}=\delta e^{-n\tau }+n,\;\;h_{*}=1. \end{array}$$

Using Taylor expansion on the right-hand side of (3) around (*k*_∗_, *h*_∗_), we get the following linearized system:

(5) k˙=−n(k−k∗)−(M−ab)k∗b(h−h∗)+M(kd−k∗) +Mk∗b(hd−h∗),h˙=−ab(h−h∗),

where

(6) $$\begin{array}{c} M=\left(\alpha -1\right)\delta e^{-n\tau }+\alpha n. \end{array}$$

The corresponding characteristic equation is of the following form:

(7) |−n−λ+Me−λτ−(M−ab)k∗b+Mk∗be−λτ0−ab−λ| =(−ab−λ)(−n−λ+Me−λτ)=0.

Equation (7) has always the root *λ* = −*ab*. Other possible roots are those solutions of

(8) $$\begin{array}{c} P\left(\lambda ,\tau \right)=-n-\lambda +Me^{-\lambda \tau }=0. \end{array}$$

Equation (8) is a transcendental equation which, in general, has an infinite number of complex roots. As usual, one begins by considering the case with no delay. In this case, it is straightforward to see that the characteristic equation (7) has only *λ* = −*ab* < 0 and *λ* = (*α* − 1)(*n* + *δ*) < 0 as roots. Then for *τ* = 0 all of the roots of the polynomial (7) have negative real part. Hence, the equilibrium point (*k*_∗_, *h*_∗_) is locally asymptotically stable in the absence of the delay. As *τ* varies, these roots change. The question is whether the equilibrium can undergo any stability switch as *τ* is increased. To identify a stability switch, we seek solutions of the characteristic equation of the form *λ* = 0 and *λ* = *iω*, *ω* ∈ ℝ.

## 3. Stability and Existence of Hopf Bifurcation

In this section, we mainly study stability and existence of limit cycle of system (3). We begin by investigating the existence of the critical stability boundary *λ* = 0. In this case, *P*(0, *τ*) = −*n* + *M* = (*α* − 1)(*n* + *δe* ^−*αnτ*^) ≠ 0 implies that *λ* = 0 is not a characteristic root of (8). Next, we look for a purely imaginary root *iω*, *ω* ∈ ℝ, of (8). Without loss of generality, one can assume *λ* = *iω*, with *ω* > 0, to be a root of (8), since the roots of (8) appear as complex conjugate pair. Therefore, one has

(9) $$\begin{array}{c} P\left(i\omega ,\tau \right)=-n-i\omega +Me^{-i\omega \tau }=0. \end{array}$$

Using Euler's identity *e* ^*iθ*^ = cos⁡*θ* + *i*sin*θ* in the above equation, and then separating the polynomial into its real and imaginary parts, we get

(10) $$\begin{array}{c} \omega =-M\sin\omega \tau ,\;\;n=M\cos\omega \tau . \end{array}$$

Squaring each equation and summing the results yield

(11) $$\begin{array}{c} \omega^{2}=M^{2}-n^{2}. \end{array}$$

A stability switch cannot occur for *τ* such that |*M*| ≤ *n*, that is, if |(*α* − 1)*δe* ^−*nτ*^ + *αn*| ≤ *n*, while it may occur as *τ* is varied when |*M*| > *n*. We have the following result.

**Lemma**

There exists as a unique positive root *ω*(*τ*) of (11), if

(12) $$\begin{array}{c} \frac{\left(1-\alpha \right)\delta }{\left(1+\alpha \right)n}>1,\;\;\tau <\frac{1}{n}\ln\left[\frac{\left(1-\alpha \right)\delta }{\left(1+\alpha \right)n}\right]. \end{array}$$

**Proof**

The condition |*M*| > *n* means |(*α* − 1)*δe* ^−*nτ*^ + *αn*| > *n*, from which we have (*α* − 1)*δe* ^−*nτ*^ + *αn* > −*n* and (*α* − 1)*δe* ^−*nτ*^ + *αn* > *n*. Therefore, we find

(13) $$\begin{array}{c} e^{-n\tau }>\frac{\left(1+\alpha \right)n}{\left(1-\alpha \right)\delta }⟹\tau <\frac{1}{n}\ln\left[\frac{\left(1-\alpha \right)\delta }{\left(1+\alpha \right)n}\right]. \end{array}$$

The conclusion hold according to the fact that the term (1 − *α*)*δ*/(1 + *α*)*n* is less or bigger than one.

We have shown that pairs of eigenvalues cross the imaginary axis as *τ* passes through certain critical values. From (10), we can derive that the critical values of *τ* and the corresponding purely imaginary eigenvalues ±*iω*(*τ*), *ω*(*τ*) > 0, where

(14) $$\begin{array}{c} \omega \left(\tau \right)=\sqrt{M^{2}-n^{2}}, \end{array}$$

are given implicitly by

(15) $$\begin{array}{c} \sin\left[\omega \left(\tau \right)\tau \right]=-\frac{\omega \left(\tau \right)}{M},\\ \cos\left[\omega \left(\tau \right)\tau \right]=\frac{n}{M}. \end{array}$$

It is impossible to solve these equations for *τ* explicitly, so we will use the procedure described in Beretta and Kuang [16]. According to this procedure, one defines *θ*(*τ*)∈(0,2*π*) as solution of (15) with *ω*(*τ*) given by (14). This defines *θ*(*τ*) in a form suitable for numerical evaluation using standard software. Then *τ* is still given implicitly by

(16) $$\begin{array}{c} \tau_{j}\left(\tau \right)=\frac{\theta \left(\tau \right)+2j\pi }{\omega \left(\tau \right)},\;j=0,1,2,\ldots . \end{array}$$

From (10), we have

(17) $$\begin{array}{c} \tau_{j}=\frac{\tan^{-1}\left[-\omega \left(\tau \right)/n\right]+\left(2j+1\right)\pi }{\omega \left(\tau \right)},\;j=0,1,2,\ldots . \end{array}$$

The occurrence of stability switches takes place at the zeros of the functions

(18) $$\begin{array}{c} S_{j}\left(\tau \right)=\tau -\tau_{j}\left(\tau \right),\;j=0,1,2,\ldots . \end{array}$$

We can locate the zero of functions *S*_*j*_(*τ*) to provide thresholds for stability switches by Maple or other popular mathematical software. With the aid of these plots, a designer can determine by a glance what values of delay *τ* must be chosen in order to have a stable system.

**Proposition**

A pair of simple conjugate pure imaginary roots *λ* = ±*iω*(*τ*_*j*_), with *ω*(*τ*_*j*_) > 0, of (8) exists at *τ*_*j*_ ∈ [0, *τ*_*c*_), if *S*_*j*_(*τ*) = 0 for some *j* = 0,1, 2,…, where

(19) $$\begin{array}{c} \tau_{c}=\frac{1}{n}\ln\left[\frac{\left(1-\alpha \right)\delta }{\left(1+\alpha \right)n}\right],\;\;\frac{\left(1-\alpha \right)\delta }{\left(1+\alpha \right)n}>1. \end{array}$$

The pair of conjugate pure imaginary roots crosses the imaginary axis from left to right if Λ(*τ*_*j*_) > 0 and crosses the imaginary axis from right to left if Λ(*τ*_*j*_) < 0, where

(20) Λ(τj)=sign⁡[d(Reλ)dτ|λ=iω(τj)]=sign⁡[dSj(τ)dτ|τ=τj].

**Proof**

From the previous Lemma, we know that the existence of *ω* = *ω*(*τ*) is guaranteed. Also, *λ* = *iω* must be a simple root for (8), otherwise *P*(*iω*, *τ*) = *P*′(*iω*, *τ*) = 0 would lead to the contradiction *iω* *τ* + *nτ* + 1 = 0. Next, differentiation of (8) with respect to *τ* gives

(21) $$\begin{array}{c} \frac{d\lambda }{d\tau }=-\frac{\left(\lambda +n\right)\left(M\lambda -M^{\prime }\right)}{M\left[1+\left(\lambda +n\right)\tau \right]}, \end{array}$$

where *M*′ = *dM*/*dτ* = (1 − *α*)*n* *δe* ^−*nτ*^. Then (21) yields

(22) Re[(dλdτ)−1]λ=iω(τj)=M[ω(τj)]2+M′(n+M2τ)M{[Mω(τj)]2+(M′)2}.

Differentiating (17) with respect to *τ*, and noting, from (11), that *ωω*′ = *MM*′, one has

(23) $$\begin{array}{c} \frac{dS_{j}\left(\tau \right)}{d\tau }=1-\frac{d\tau_{j}}{d\tau }=\frac{M\omega^{2}+M^{\prime }\left(n+M^{2}\tau_{j}\right)}{M\omega^{2}}. \end{array}$$

We remark that

(24) sign⁡[d(Reλ)dτ]=sign⁡[Re(dλdτ)−1].

Hence, comparing (23) evaluated at *τ* = *τ*_*j*_ with (22) leads to the conclusion.

We have seen that the couple of simple pure imaginary roots of (8),  *λ* = ±*iω*(*τ*), *ω*(*τ*) > 0 solution of (11), occur at the *τ* values which are the zeros of the functions *S*_*j*_ in (18). In the light of the previous analysis and recalling that (*k*_∗_, *h*_∗_) is locally asymptotically stable when *τ* = 0, certain conclusions follow.

**Theorem**

Let *τ*_*c*_ > 0 be defined as in the previous proposition.

1. The positive equilibrium (*k*_∗_, *h*_∗_) of (3) is locally asymptotically stable for all *τ* ≥ 0 if the following condition: (1 − *α*)*δ* / [(1 + *α*)*n*] ≤ 1, holds and for all *τ*≥*τ*_*c*_ otherwise.
2. For all <*τ*_*c*_, if *S*_*j*_(*τ*), *j* = 0,1, 2,…, have positive simple zeros, say *τ*_1_ < *τ*_2_ < ⋯<*τ*_*N*_, then (3) undergoes a Hopf bifurcation at (*k*_∗_, *h*_∗_) when *τ* = *τ*_*j*_, *j* = 1,…, *N*. Moreover, the equilibrium (*k*_∗_, *h*_∗_) may change its stability finitely many times through stability switches.

## 4. Stability and Direction of the Hopf Bifurcation

In the previous section, we have obtained conditions which guarantee that system (3) undergoes the Hopf bifurcation at the critical values *τ*_*j*_. In this section, we will study the direction, stability, and the period of the bifurcating periodic solutions, based on the normal form approach and the center manifold theory introduced by Hassard et al. [17]. For notation convenience, let *τ* = *τ*_*j*_ + *μ*, where *μ* ∈ ℝ. Then *μ* = 0 is the Hopf bifurcation value for system (3) in terms of the new bifurcation parameter *μ*. Set *u* = (*u*_1_, *u*_2_) = (*k* − *k*_∗_, *h* − *h*_∗_). Rescaling the time by *t* → *t* / *τ* to normalize the delay, system (3) can be written as a functional differential equation in the phase space *C*([−1,0], ℝ^2^). Applying Taylor expansion to the right-hand side of system (3) at the equilibrium point and then separating the linear from the nonlinear terms, system (3) becomes

(25) $$\begin{array}{c} \dot{u}=L_{\mu }\left(u_{t}\right)+f\left(\mu ,u_{t}\right), \end{array}$$

where *u*_*t*_ ∈ *C*([−1,0], ℝ^2^), *u*_*t*_ = *u*(*t* + *θ*), for *θ* ∈ [−1,0], and the maps *L*_*μ*_ : *C*([−1,0], ℝ^2^) → ℝ^2^ and *f* : ℝ × *C*([−1,0], ℝ^2^) → ℝ^2^ are defined as follows:

(26) Lμ(φ)=(τj+μ)[−n−(M−ab)k∗b0−ab]φ(0) +(τj+μ)[MMk∗b00]φ(−1),

(27) $$\begin{array}{c} f\left(\mu ,\varphi \right)=\left(\tau_{j}+\mu \right)\left[\begin{array}{c} f^{\left(1\right)}\\ f^{\left(2\right)} \end{array}\right], \end{array}$$

where *φ* = (*φ*_1_, *φ*_2_) ∈ *C*([−1,0], ℝ^2^). Let

(28) P(k,kd,h,hd)=se−αnτ[(hdh−1)]α / bkdα−δe−nτ(hdh−1)1 / bkd −[n+a(1−h)]k.

Then the nonlinear parts *f* ^(1)^, *f* ^(2)^ are given by

(29)  f(1)=12[Pkdkd∗φ1(−1)2+Phh∗φ2(0)2+2Pkdh∗φ1(−1)φ2(0)+2Pkh∗φ1(0)φ2(0)] +13![Pkdkdkd∗φ1(−1)3+Phhh∗φ2(0)3+3Pkdkdh∗φ1(−1)2φ2(0)+3Pkdhh∗φ1(−1)φ2(0)2]+⋯,f(2)=(−ab)φ2(0)2,

where

(30) Pkdkd∗=α(α−1)(δe−nτ+n)k∗−1,Phh∗=[(1+α/b)αn+(1+(1+α)/b)(α−1)δe−nτ]k∗b,Pkdh∗=(1−α2)δe−αnτ−α2nb,  Pkh∗=a,Pkdkdkd∗=(α−2)k∗−1Pkdkd∗,Phhh∗=([−(2+αb)(1+αb)(δe−nτ+n)α+(2+1b)(1+1b)δe−nτ]k∗)×(b)−1,Pkdkdh∗=−αbPkdkd∗,Pkdhh∗=[(1+α/b)(δe−nτ+n)α2−(1+1/b)δe−nτ]b.

Here, we use the notation *P*_jl_* = *P*_*jl*_(*k*_∗_, *h*_∗_), *j*, *l* ∈ {*k*, *k*_*d*_, *h*, *h*_*d*_}.

By using the Riesz representation theorem, there exists a matrix whose components are bounded variation function *η*(*θ*, *μ*) for *θ* ∈ [−1,0] such that

(31) $$\begin{array}{c} L_{\mu }\varphi =\int_{-1}^{0}d\eta \left(\theta ,\mu \right)\varphi \left(\theta \right),\;\text{for}\;\varphi \in C\left(\left[-1,0\right],{\mathbb{R}}^{2}\right). \end{array}$$

In fact, we can choose

(32) η(θ,μ)={(τj+μ)[−n−(M−ab)k∗b0−ab]Γ(θ)  +(τj+μ)[MMk∗b00]Γ(θ+1)},

where Γ denotes the Dirac delta function. For *φ* ∈ *C*([−1,0], ℝ^2^), define

(33) A(μ)(φ)={dφ(θ)dθ,θ∈[−1,0),∫−10dη(r,μ)φ(r),θ=0,R(μ)(φ)={0,θ[∈−1,0),f(μ,φ),θ=0.

Then system (25) is equivalent to the following system of ordinary differential equations:

(34) $$\begin{array}{c} \dot{u_{t}}=A\left(\mu \right)u_{t}+R\left(\mu \right)u_{t}, \end{array}$$

where *u*_*t*_ = *u*(*t* + *θ*), for *θ* ∈ [−1,0]. For *ψ* ∈ *C*([0,1], ℝ^2^), define

(35) A∗ψ(r)={−dψ(r)dr,r∈(0,1],∫−10dηT(ζ,μ)ψ(−ζ),r=0,

and a bilinear inner product

(36) 〈ψ(r),φ(θ)〉  =ψ¯(0)φ(0)−∫θ=−10∫ξ=0θψ¯(ξ−θ)dη(θ)φ(ξ)dξ,

where *η*(*θ*) = *η*(*θ*, 0). Then *A*(0) and A* are adjoint operators. In order to determine the Poincaré normal form of the operator *A*(0), we need to calculate the eigenvector *q*(*θ*) of *A*(0) corresponding to the eigenvalue *iω*(*τ*_*j*_)*τ*_*j*_ and the eigenvector q*(*r*) of A* corresponding to the eigenvalue −*iω*(*τ*_*j*_)*τ*_*j*_. Suppose that *q*(*θ*) = (1, *ρ*)^*T*^ *e* ^*iω*(*τ*_*j*_)*τ*_*j*_*θ*^, with *ρ* complex, is the eigenvector of *A*(0) corresponding to *iω*(*τ*_*j*_)*τ*_*j*_. Since *A*(0)*q*(*θ*) = *iω*(*τ*_*j*_)*τ*_*j*_ *q*(*θ*), then it follows from the definition of *A*(0), (31), and (32) that we can derive *q*(0) = (1, *ρ*)^*T*^. Similarly, supposing that q*(*r*) = *D*(*σ*, 1)*e* ^*iω*(*τ*_*j*_)*τ*_*j*_*r*^ is the eigenvector of A* corresponding to −*iω*(*τ*_*j*_)*τ*_*j*_, we can get q*(0), with the value of *D* chosen to guarantee that 〈q*, *q*〉 = 1.

In the remainder of this section, we will follow the ideas and the same notations as in Hassard et al. [17] and compute the coordinates to describe the center manifold *𝒞* at *μ* = 0. Let *u*_*t*_ be the solution of (34) when *μ* = 0. Define

(37) z=〈q∗,ut〉,  W(t,θ)=ut(θ)−2Re{zq(θ)}.

On the center manifold, one has

(38) W(t,θ)=W(z,z¯,θ)=W20(θ)z22+W11(θ)zz¯+W02(θ)z¯22+⋯,

where *z* and $\bar{z}$ are local coordinates for the center manifold in the direction of q* and ${\bar{q}}^{*}$. Noticing that *W* is real if *u*_*t*_ is real, we consider only real solution. For the solution *u*_*t*_ ∈ *𝒞*, as *μ* = 0, from (37) we have

(39) z˙=iω(τj)τjz+q¯∗(0)f(0,W(z,z¯,0)2Re{zq(0)})≝iω(τj)τjz+q¯∗(0)f0(z,z¯),

where $f_{0}(z,\bar{z})=f(0,u_{t})$, with *f* defined as in (27). Denote ${\bar{q}}^{*}(0)f_{0}(z,\bar{z})$ by $g(z,\bar{z})$. Writing the Taylor expansion, we have

(40) g(z,z¯)=q¯∗(0)f0(z,z¯)=g20z22+g11zz¯+g02z¯22 +g21z2z¯2+⋯.

From (37), we get

(41) ut(θ)=W(t,θ)+2Re{zq(θ)}=W20(θ)z22+W11(θ)zz¯ +W02(θ)z¯22+⋯+zq(θ)+z¯ q¯(θ).

Substituting into *f*(0, *u*_*t*_) yields

(42) f0(z,z¯)=f(0,ut)=fz2z22+fzz¯zz¯+fz¯2z¯22+fz2z¯z2z¯2+⋯.

Comparing the coefficients of (42) with those in (40), we find

(43) g20=D¯(σ¯,1)fz2,  g02=D¯(σ¯,1)fz¯2,g11=D¯(σ¯,1)fzz¯,  g21=D¯(σ¯,1)fz2z¯.

The term *g*_21_ is dependent on *W*_*jl*_ (*j* + *l* = 2). Hence, in the sequel, we will compute them. From (25) and (37), we have

(44) W˙=ut˙−z˙q−z¯ ˙q¯={AW−2Re{q¯∗(0)f0q(θ)},θ∈[−1,0),AW−2Re{q¯∗(0)f0q(0)}+f0,θ=0,≝AW+H(z,z¯,θ),

where

(45) $$\begin{array}{c} H\left(z,\bar{z},\theta \right)=H_{20}\left(\theta \right)\frac{z^{2}}{2}+H_{11}\left(\theta \right)z\bar{z}+H_{02}\left(\theta \right)\frac{{\bar{z}}^{2}}{2}+\cdots . \end{array}$$

Expanding the above series and comparing the corresponding coefficients, we obtain

(46) [A−2iω(τj)τjI]W20(θ)=−H20(θ),AW11(θ)=−H11(θ).

By (44), we know that, for *θ* ∈ [−1,0),

(47) H(z,z¯,θ)=−q¯∗(0)f0q(θ)−q∗(0)f¯0q¯(θ)=−gq(θ)−g¯ q¯(θ).

Comparing the coefficients with (45),

(48) H20(θ)=−g20q(θ)−g¯02q¯(θ),H11(θ)=−g11q(θ)−g¯11q¯(θ).

From (47), (48), and the definition of *A*,

(49) $$\begin{array}{c} {\dot{W}}_{20}\left(\theta \right)=2i\omega \left(\tau_{j}\right)\tau_{j}W_{20}\left(\theta \right)+g_{20}q\left(\theta \right)+{\bar{g}}_{02}\bar{q}\left(\theta \right). \end{array}$$

Recalling that *q*(*θ*) = (1, *ρ*)^*T*^ *e* ^*iω*(*τ*_*j*_)*τ*_*j*_*θ*^ and solving the previous equation for *W*_20_(*θ*), one has

(50) W20(θ)=−g20iω(τj)τjq(0)eiω(τj)τjθ −g¯023iω(τj)τjq¯(0)e−iω(τj)τjθ+E1e2iω(τj)τjθ,

where *E*_1_ = (*E*_1_^(1)^, *E*_1_^(2)^) ∈ ℝ^2^ is a constant vector. Similarly, again from (47) and (48), we can derive

(51) W11(θ)=g11iω(τj)τjq(0)eiω(τj)τjθ −g¯11iω(τj)τjq¯(0)e−iω(τj)τjθ+E2,

where *E*_2_ = (*E*_2_^(1)^, *E*_2_^(2)^) ∈ ℝ^2^. In the following, we will seek for *E*_1_ and *E*_2_. From the definition of *A* and (46), we have

(52) $$\begin{array}{c} \int_{-1}^{0}d\eta \left(\theta \right)W_{20}\left(\theta \right)=2i\omega \left(\tau_{j}\right)\tau_{j}W_{20}\left(\theta \right)-H_{20}\left(\theta \right), \end{array}$$

(53) $$\begin{array}{c} \int_{-1}^{0}d\eta \left(\theta \right)W_{11}\left(\theta \right)=-H_{11}\left(\theta \right). \end{array}$$

From (44) and (45), we have

(54) H20(0)=−g20q(0)−g¯02q¯(θ)+fz2,  H11(0)=−g11q(0)−g¯11q¯(0)+fzz¯.

Substituting (50) and (54) into (52) and noticing that

(55) $$\begin{array}{c} \left[i\omega \left(\tau_{j}\right)\tau_{j}I-\int_{-1}^{0}e^{i\omega (\tau_{j})\tau_{j}\theta }d\eta \left(\theta \right)\right]q\left(0\right)=0,\\ \left[-i\omega \left(\tau_{j}\right)\tau_{j}I-\int_{-1}^{0}e^{-i\omega (\tau_{j})\tau_{j}\theta }d\eta \left(\theta \right)\right]\bar{q}\left(0\right)=0, \end{array}$$

we obtain

(56) $$\begin{array}{c} \left[2i\omega \left(\tau_{j}\right)\tau_{j}I-\int_{-1}^{0}e^{2i\omega (\tau_{j})\tau_{j}\theta }d\eta \left(\theta \right)\right]E_{1}=f_{z^{2}}. \end{array}$$

Similarly, from (51) and (53), we can get

(57) $$\begin{array}{c} \left[\int_{-1}^{0}d\eta \left(\theta \right)\right]E_{2}=f_{z\bar{z}}. \end{array}$$

These show that *E*_1_ and *E*_2_ can be determined. Based on the above analysis, each *g*_*ij*_ is computed. Therefore, we can calculate the following quantities:

(58) c1(0)=i2ω(τj)τj[g11g20−2|g11|2−|g02|23]+g212,μ2=−Re{c1(0)}Re{τjλ′(τj)},  β2=2Re{c1(0)},T2=−Im⁡{c1(0)}+μ2Im⁡{λ′(τj)}ω(τj)τj,

which determine the properties of bifurcating periodic solutions. Specifically, *μ*_2_, *β*_2_, and *T*_2_ determine the direction, stability, and period of the corresponding Hopf bifurcation, respectively.

**Theorem**

(1) The direction of the Hopf bifurcation of the system (3) at the equilibrium (*k*_∗_, *h*_∗_) when *τ* = *τ*_*j*_ is subcritical (resp., supercritical) *μ*_2_ < 0 (resp., *μ*_2_ > 0); that is, there exists a bifurcating periodic solution for *τ* < *τ*_*j*_ (resp., *τ* > *τ*_*j*_) in the sufficiently small *τ*_*j*_-neighbourhood.

(2) The bifurcating periodic solution on the center manifold is unstable (resp., locally asymptotically stable), if *β*_2_ > 0 (resp., *β*_2_ < 0).

(3) The period of the bifurcating periodic solution decreases (resp., increases), if *T*_2_ < 0 (resp., *T*_2_ > 0).

## 5. Numerical Simulations

In this section, we study how the long-run dynamics of the dynamical system (3) change when the time delay parameter varies. The configuration of parameters is the following: *a* = .6, *b* = .4, *n* = .0075, *α* = .43, *δ* = .09, and (1 − *α*)*δ* / [(1 + *α*)*n*] > 1.

**Figure 1:**
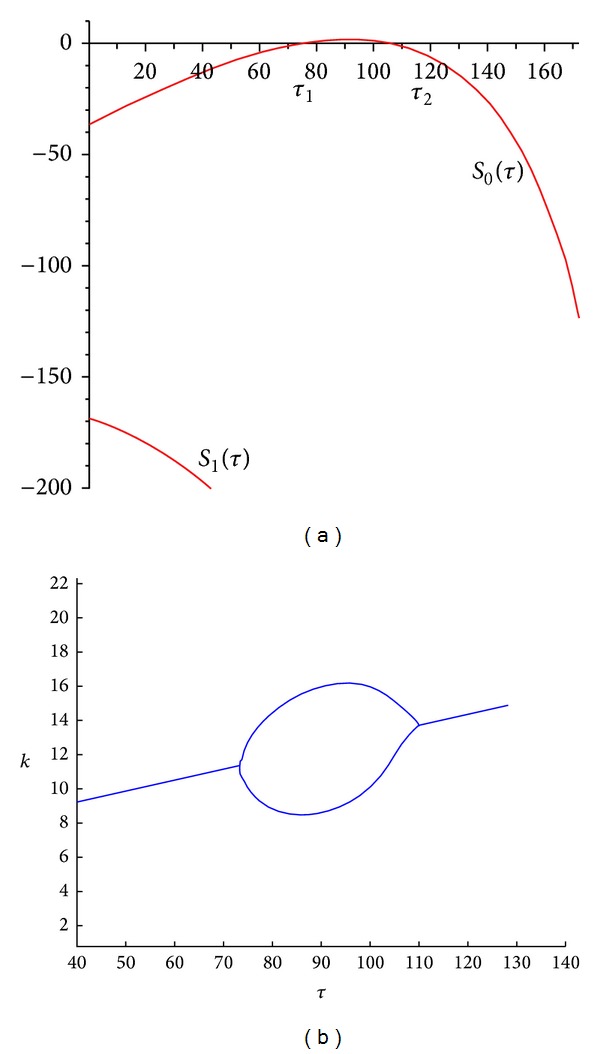
(a) Graph of stability switch in terms of time delay for the system (3). *τ*_1_≅74.22, *τ*_2_≅106.43; (b) bifurcation diagram.

Figure 1(a) shows the behaviour of *S*_0_(*τ*) and *S*_1_(*τ*): there exist *τ*_1_ and *τ*_2_ such that (*k*_∗_, *h*_∗_) is locally stable for *τ* < *τ*_1_ and *τ* > *τ*_2_ while oscillations exist for *τ* ∈ (*τ*_1_, *τ*_2_).  Figure 1(b) depicts the maximum and minimum value assumed by *k* with respect to the bifurcation parameter *τ*.

**Figure 2:**
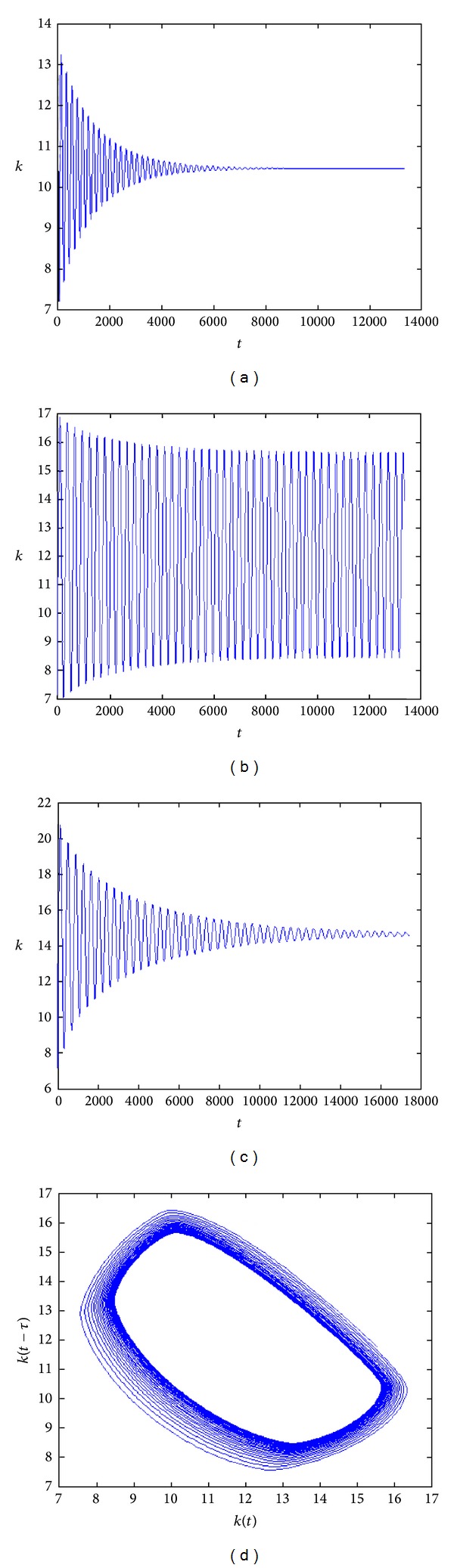
(c) The time evolution of the function *k*(*t*) for *τ* = 60 (a); *τ*≅87 (b); *τ* = 120; (d) attracting limit cycle in the plane *k*(*t*), *k*(*t* − *τ*).

The time series of capital stock according to different values of the time delay are shown in Figure 2. In particular, Figure 2(b) describes endogenous oscillations (not driven by stochastic shocks) typical of real world economic variables.

## 6. Conclusions

We have analysed the dynamical properties of a Solow model with bounded technological progress and time-to-build technology. We have shown that the introduction of these two components drastically changes the results of the classical models with exponential growth of technological progress or without delays. In particular, varying the time delay, the system is able to produce stability switches and Hopf bifurcations. Since problems concerning economic growth and knowledge accumulation are usually studied on BGP (balanced growth path) or when the steady state of the model is stable, we believe that our dynamical analysis may be useful to understand the short run fluctuations of the economic dynamics in a theoretical model. Some possible extensions of the present analysis should also be mentioned. First, a more general structure of time delays may be introduced (different delays for technological and physical productive factors). Second, the allocative process may be endogenized.
